# Optimised treatment of patients with enlarged lateral lymph nodes in rectal cancer: protocol of an international, multicentre, prospective registration study after extensive multidisciplinary training (LaNoReC)

**DOI:** 10.1136/bmjopen-2023-083225

**Published:** 2024-10-16

**Authors:** Eline G M van Geffen, Tania C Sluckin, Sanne-Marije J A Hazen, Karin Horsthuis, Martijn Intven, Susan van Dieren, Geerard Beets, Marilyne M Lange, Melissa W Taggart, Regina G H Beets-Tan, Corrie A M Marijnen, Tsuyoshi Konishi, Pieter J Tanis, Miranda Kusters, The LaNoReC study group, Marc R. W. Engelbrecht, Elisabeth D. Geijsen, Roel Hompes, Philip Meijnen, Jaap Stoker, Jurriaan B. Tuynman, Ingrid M. Bruijnzeel, Bas Lamme, Femke M. Alberts, Peter A. Neijenhuis, Rogier M. P. H. Crolla, Joanne Verdult, Johan H. Wijsman, Charlotte S. van Kessel, Erik Jan Mulder, Jan Binne Hulshoff, Ivan M. Cherepanin, Hans F. J. Fabry, G. Y. Mireille, Frank J. M. Kemper, Fatih Polat, Johanne G. Bloemen, Jacobus W. A. Burger, Jeltsje S. Cnossen, Joost Nederend, Shira H. de Bie, Robbert J. I. Bosker, Aaldert K. Talsma, Leonora S. F. Boogerd, Marc J. P. M. Govaert, Merel M. Scheurkogel, Imogeen E. Antonisse, Michail Doukas, Joost Rothbarth, Marianne de Vries, Marcel A. H. Ribbert, Anthony W. H. van de Ven, Susan ter Borg, Jennifer W. Bradshaw, Maria Verseveld, Heleen M. Ceha, Fleur I. de Korte, Andreas W. K. S. Marinelli, Marinke Westerterp, Tjeerd S. Aukema, Liselotte W. van Bockel, Aukje A. J. M. van Tilborg, Tom Rozema, Amarins Brandsma, Stefan Hoogendoorn, Saskia R. Offerman, Hanneke Vos, Henderik L. van Westreenen, Jeroen W. A. Leijtens, Fabian A. Holman, Koen C.M.J. Peeters, Laura A. Velema, L Els, van Persijn van Meerten, Frans C. H. Bakers, Jarno Melenhorst, Iryna Samarska, Nina Šefčovičová, Maaike Berbée, Bastiaan B. Pultrum, Dennis B. Rouw, Matthew Albert, L. René Arensman, Hanneke Basart, Esther C. J. Consten, Bart C.T. van de Laar, Inne Somers, Paul M. Verheijen, Thomas A. Fassaert, Christiaan Hoff, Eino B. van Duyn, Ellen M. Hendriksen, Hugo A.J. Gielkens, Arend G. J. Aalbers, Brechtje A. Grotenhuis, Michalda S. Dunker, Anne M. van Geel, Christof Meischl, W. Hermien Schreurs, Patty H. Spruit, Michael F. Gerhards, Thomas M. Karsten, Eveline J.T. Krul, Jan Peringa, Sebastiaan van Koeverden, Andre J. A. Bremers, Heidi Rütten, Johannes H. W. de Wilt, Mariska den Hartogh, Karin Muller, Vera Oppedijk, Jan Willem T. Dekker, Debora Eschbach-Zandbergen, Krista Gerbrands, Daphne Roos, Arjan van Tilburg, Ernst Jan, Spillenaar Bilgen, Nikki Knijn, Marnix A. J. de Roos, Ilse van Dop, Michael Croft, Tracy Fitzsimmons, Hidde M. Kroon, Michael Penniment, Mitchell Raeside, Andrew Ruszkiewicz, Tarik Sammour, Michael Wilks, Steven J. Oosterling, Jeroen A. W. Tielbeek, Ronald J. C. L. M. Vuylsteke, Erik J. R. J. van der Hoeven, Anke B. Smits, Anniek H. Boer, Edgar J. B. Furnée, Robbert J. de Haas, Klaas Havenga, Manon N. G. J. A. Braat, Wilhelmina M. U. van Grevenstein, Milan C. Richir, Patricia J. A. M. Brouwers, Tilly Leseman, Eric H. J. Belgers, Evert-Jan G. Boerma, Jasenko Krdzalic, Robert Riedl, Roy F. A. Vliegen

**Affiliations:** 1 Surgery, Amsterdam UMC Location VUmc, Amsterdam, North-Holland, the Netherlands; 2 Treatment and Quality of Life, Imaging and Biomarkers, Amsterdam, Cancer Center Amsterdam, Amsterdam, the Netherlands; 3 Radiology, Amsterdam UMC Location VUmc, Amsterdam, North-Holland, the Netherlands; 4 Radiation Oncology, University Medical Centre Utrecht, Utrecht, Utrecht, the Netherlands; 5 Surgery, Amsterdam UMC Location AMC, Amsterdam, North-Holland, the Netherlands; 6 Surgical Oncology, Antoni van Leeuwenhoek, the Netherlands Cancer Institute, Amsterdam, North-Holland, the Netherlands; 7 Pathology, Amsterdam UMC Location AMC, Amsterdam, North-Holland, the Netherlands; 8 Pathology, MD Anderson Gastrointestinal Cancer Center, Houston, Texas, USA; 9 Radiology, Antoni van Leeuwenhoek, the Netherlands Cancer Institute, Amsterdam, North-Holland, the Netherlands; 10 Radiation Oncology, Antoni van Leeuwenhoek, the Netherlands Cancer Institute, Amsterdam, North-Holland, the Netherlands; 11 Surgery, MD Anderson Gastrointestinal Cancer Center, Houston, Texas, USA; 12 Surgery, Erasmus Medical Center, Rotterdam, South-Holland, the Netherlands

**Keywords:** Patients, Colorectal surgery, Gastrointestinal tumours, Patient Reported Outcome Measures

## Abstract

**Introduction:**

Inadequate treatment of enlarged lateral lymph nodes (LLNs) in rectal cancer patients is associated with an increased lateral local recurrence (LLR) risk, despite neoadjuvant treatment and total mesorectal excision (TME) surgery. There is a promising role for LLN dissection (LLND) to lower this risk, but this challenging procedure requires appropriate training. This study protocol describes a prospective evaluation of oncological outcomes after standardised treatment based on multidisciplinary training, thereby aiming for a 50% reduction in LLR rate.

**Methods and analysis:**

A prospective registration study will be opened in hospitals in which the involved multidisciplinary team members (radiologists, radiation oncologists, surgeons and pathologists) have received dedicated training to enhance knowledge and awareness of LLNs and in which standardised treatment including LLND has been implemented. Patients with rectal cancer and at least one enlarged LLN (short-axis ≥7.0 mm), or intermediate LLN (short-axis 5.0–6.9 mm) with at least one malignant feature on primary MRI, evaluated by a trained radiologist, are eligible. Patients will undergo neoadjuvant treatment by trained radiation oncologists, followed by TME surgery in combination with a minimally invasive, nerve-sparing LLND performed by trained surgeons. LLND specimens are evaluated by trained pathologists or grossing assistants. The primary outcome is LLR rate 3 years postoperatively. Secondary outcomes are morbidity, disease-free survival, overall survival and quality of life. To demonstrate a significant reduction in LLR rate from 13% (based on historical control data) to 6% after optimised treatment, 200 patients with enlarged LLNs are required.

**Ethics and dissemination:**

The medical ethics board of the Vrije Universiteit Medical Centre (VUMC), the Netherlands, approved the study on 23 November 2022 (reference: 2021.0524). Participating centres must obtain local approval and participants are required to provide written informed consent. Results obtained from this study will be communicated via peer-reviewed medical journals and presentations at conferences.

**Trail registration number:**

NCT04486131, 24 July 2020, https://clinicaltrials.gov/ct2/show/NCT04486131.

**STRENGTHS AND LIMITATIONS OF THIS STUDY**

- The lateral nodal recurrence in rectal cancer (LaNoReC) study is grounded on multidisciplinary training of all specialties involved in the treatment of locally advanced rectal cancer.
- Monthly meetings for quality control will discuss all new inclusions and surgical lateral lymph node dissection videos, thereby increasing quality and standardisation of eligibility criteria and subsequent treatment.
- The LaNoReC study will gather data on quality of life and morbidity/functional outcomes, providing novel insights into these aspects of care in Western patients.
- The non-randomised design is a risk for heterogeneity in treatments but has been chosen due to expected difficult accrual if a specific treatment protocol is imposed and the inability to properly define the control treatment.

## Introduction

 The last few decades have witnessed major improvements in rectal cancer treatment due to the use of MRI for adequate staging, neoadjuvant treatment and the total mesorectal excision (TME) procedure, as reflected by a decrease in the overall local recurrence (LR) rate to 5%–10%.^1^,^3^ Consequently, occurrence of LRs has shifted from a central location to the lateral pelvic compartments, the latter now comprising approximately half of all LRs.^4^ Most likely, these are caused by lymphatic spread to lateral lymph nodes (LLNs) in locally advanced rectal cancer (LARC) in the lower part of the rectum.^4^ When these LLNs are primarily enlarged (short-axis ≥7.0 mm), the percentage of lateral LR (LLR) has been reported to be up to 20% after 5 years following neoadjuvant (chemo)radiotherapy (C)RT and TME.^5^,^7^ LLRs are associated with fewer complete resections, and consequently, poorer survival outcomes compared with recurrences at other sites.^8 9^

Different approaches are used worldwide to treat enlarged LLNs. Eastern surgeons, particularly from Japan, perform an LLN dissection (LLND) to decrease the risk of LLR,^10^ despite the associated morbidity.^11 12^ However, the generalisability of studies supporting LLND’s effectiveness is limited due to exclusions of patients with LLNs ≥10.0 mm and the differences in population and practice between the East and the West.^13^ In the West, physicians primarily rely on neoadjuvant (C)RT to treat enlarged LLNs, or they are erroneously considered as systemic disease, leading to undertreatment resulting in high LLR rates.^11^

With recent publications, awareness concerning the importance of LLNs has gradually increased in the West. The international multicentre study by the Lateral Node Consortium found an increased LLR rate of up to 20% after 5 years for primarily enlarged LLNs (≥7.0 mm), which could be significantly lowered to a 3-year LLR rate of 6% with selective LLND after (C)RT.^6^ A second study, the Snapshot Rectal Cancer 2016 study, included 3057 patients operated on for rectal cancer in 2016 in 67 Dutch centres and demonstrated a 3-year LLR rate of 11% (4 years 15%) for patients with enlarged LLNs after (C)RT+TME.^14^ While some other studies remained inconclusive about the influence of malignant features,^5 15^ that study demonstrated additional prognostic value for the presence of malignant features (round shape, irregular margins, loss of fatty hilum and heterogeneity) in intermediate size LLN (short-axis 5.0–6.9 mm), as presence increases the LLR risk from 2% to 8%.^14 16^ Moreover, in both the Consortium and the Snapshot study, LLN enlargement was not associated with increased distant metastases rates, indicating that enlarged LLNs should be considered a locoregional disease, requiring additional treatment.^11 17^

To further increase awareness and knowledge about lateral nodal disease, the Dutch radiologists, radiation oncologists and surgeons participated in training sessions.^18 19^ Radiologists followed a training session provided by expert radiologists, resulting in better short-axis measurements and anatomical classification of LLNs in cases assessed after the training versus baseline assessment.^20^ Moreover, radiation oncologists participated in several consensus meetings in which delineation variations in a similar case were discussed, leading to a renewed delineation guideline with more specific anatomical landmarks, which guarantees adequate coverage of the lateral compartments. These radiation oncologists also examined the radiotherapy delineations and doses for all LLNs with a short-axisv≥5.0 mm from the Snapshot 2016 study and demonstrated adequate coverage still being associated with considerable LLR risk and that a boost may also not prevent LLR.^21^ This suggests that any additional reduction of LLR rates may only be achieved with LLND surgery.^5 6^

In the Snapshot study, the Dutch surgeons performed node-picking and partial LLNDs, associated with increased LLR rates up to 19%.^22^ Due to a lack of experience, these procedures were sometimes performed together with urologists or gynaecologists.^23 24^ However, both specialties perform such lymphadenectomies for malignancies in the anterior pelvic compartment, and dissect mostly external iliac and the proximal part of the obturator area, while rectal cancer predominantly spreads to the distal part of the obturator and internal iliac compartment. These observations imply that formal LLND, following anatomical borders and resecting both complete obturator and internal iliac compartments, is necessary to successfully decrease the recurrence rates. Since enlarged LLNs are relatively uncommon, this procedure has been centralised in the Netherlands after several consensus meetings organised by the Dutch Society of Colorectal Surgery and 16 surgeons were nominated to follow two hands-on cadaver training sessions to get acquainted with the LLND procedure, with monthly video meetings and proctoring sessions thereafter to ensure further quality improvement.

Due to the retrospective nature of the Consortium and Snapshot studies, neither was able to properly investigate a setting in which all specialties involved are carefully trained and where a standardised formal LLND is performed, after which the uniform clearance of all LLNs within anatomical boundaries can be guaranteed. Therefore, the LaNoReC study is designed to investigate whether adequate recognition and reporting of LLNs by trained radiologists, adequate irradiation by trained radiation oncologists and performing standardised LLND with sufficient training, will successfully decrease LLR rates to below 6% for patients with enlarged LLNs. Moreover, it will evaluate current treatment strategies in patients with intermediate LLNs with malignant features.

### Methods and analysis

### Objectives

The primary objective is to evaluate whether optimisation of multidisciplinary care and nerve-sparing LLND surgery can reduce 3-year LLR rate to below 6% in rectal cancer patients with enlarged LLNs.^6^ The main secondary objectives include distant metastases rate, disease-free survival, overall survival and quality of life after 3 years. Moreover, current treatment strategies for patients with intermediate LLNs with one or more malignant features will be investigated.

### Study design

**Figure 1:**
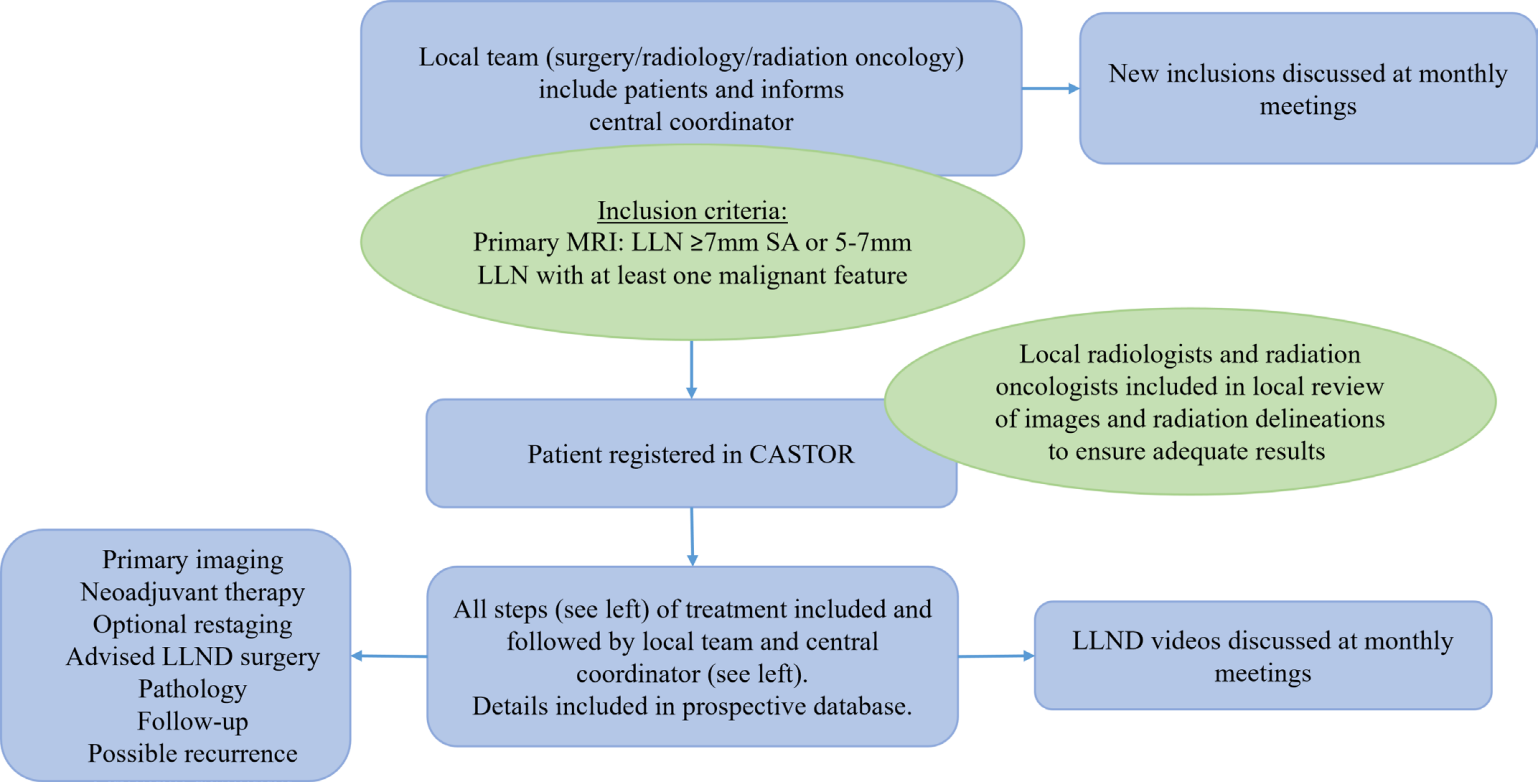
Flowchart outline of study. CASTOR, an electronic system to safely collect medical data in case report forms; LLN, lateral lymph node; LLND, lateral lymph node dissection; SA, short-axis.

The LaNoReC study is an international multicentre prospective registration study endorsed by the Dutch Colorectal Cancer Group. This study started in 2021 but was temporarily paused due to a major protocol revision. The initial protocol imposed a specific treatment (C(RT) without a boost, restaging MRI and LLND), which would have a major impact on accrual. Therefore, the protocol evolved towards the current prospective registration study, as demonstrated in figure 1. Approximately 38 Dutch hospitals, in which the multidisciplinary team has sufficed the training demands, will be open to include patients. International centres can join after following similar training sessions as the Dutch clinicians, which are organised by the LaNoReC study team. All included patients will undergo optimised standard of care, including MRI assessment by a trained radiologist, neoadjuvant radiotherapy delineation by a trained radiation oncologist, nerve-sparing LLND by a trained surgeon and evaluation of the resection specimen by a trained pathologist or grossing assistant. The study is registered at clinicaltrial.gov: NCT04486131.

### Study population

Eligible patients have a biopsy-proven adenocarcinoma of the rectum and at least one enlarged LLN of ≥7.0 mm (SA) irrespective of other features, located in a lateral compartment (internal iliac or obturator) or at least one intermediate LLN of 5.0–6.9 mm (SA) in combination with one or more malignant features (round shape, irregular margins, loss of fatty hilum or heterogeneity) on primary MRI.

Patients are not eligible if they are younger than 18 years old, have undergone previous pelvic radiotherapy, have previously undergone an LLND for a pelvic malignancy, have synchronous distant metastases, have synchronous stage III/IV colon cancer, have had another malignancy in the previous 3 years which could affect oncological outcomes (these patients must first be discussed with the research team to determine inclusion or exclusion), have an absolute contraindication for general anaesthesia, have familial adenomatous polyposis or are pregnant.

### Enrolment

Patients will be recruited based on primary MRI by the local multidisciplinary team (MDT) during an MDT meeting. The imaging of possibly eligible patients will be discussed in a monthly central meeting with the study team and local investigators from other study sites or anonymously in a coded and secured app (Siilo from Doctolib). When eligibility is confirmed, all patients will be required to provide written informed consent (online supplemental file A).

### MRI assessment by trained radiologists

Primary MRI scans are evaluated by the local radiologists and assessed according to online supplemental file B. These radiologists have completed an interactive training provided by expert radiologists from the LaNoReC study group, where the lateral compartments are discussed with specific attention to classifying the anatomical location and accurate measurements of LLNs.^14^ During the study, local radiologists will have access to two visual atlases illustrating the lateral compartments and an instruction manual to reassure the accurate diagnostics and uniform reporting of LLNs. Additional imaging will be performed according to local guidelines. For inclusion in this study, a restaging MRI after neoadjuvant treatment is advised; however, treatment decisions should be based on the primary LLN size. A restaging MRI is not obligatory but is already a standard practice in many centres. A follow-up MRI 2 years postoperatively is advised for the detection of LLR.

### Neoadjuvant radiotherapy delineation by trained radiation oncologists

Radiotherapy will take place by radiation oncologists who have completed training regarding the delineations of the lateral compartments. The delineations of the lateral compartments of the first three included patients from each centre will be evaluated by an expert radiation oncologist from the LaNoReC study team to ensure adequate coverage. Delineation of the lateral compartment and visible lymph nodes should be performed according to the renewed Dutch association of radiotion oncologists guideline (Landelijk Platform Radiotherapie Gastro-Entrerologische tumoren (LPRGE), (online supplemental file C)) to ensure adequate coverage of the lateral compartments.

In accordance with the registration design of this study, patients will undergo neoadjuvant therapy schedules according to local guidelines decided by their multidisciplinary team.^25^ These neoadjuvant options include (C)RT or total neoadjuvant treatment regimens including radiotherapy such as RAPIDO (the Rectal cancer And Preoperative Induction therapy followed by Dedicated Operation), which consists of short course radiotherapy followed by six courses of systemic chemotherapy^26^, or systemic chemotherapy combined with CRT. Based on the most recent literature, it is not recommended to give LLNs an additional irradiation boost due to limited oncological advantages and increased risks.^21^ Nevertheless, boosting of LLNs is allowed as part of the study treatment.

### Nerve-sparing LLND procedure by trained surgeons

LLNDs will only be performed by a select team of surgeons after completing adequate human cadaver training, intensive proctoring and assessment of procedural videos. Only surgeons who participated in this training can be nominated with their hospital to act as referral centre or the surgeons can be invited to perform the resection in the (local) referring centre. All ‘study-LLND’ procedure videos will be discussed during monthly meetings for quality control.

Patients with primarily enlarged (≥7.0 mm SA) LLNs are advised to undergo an LLND. Moreover, LLND should be considered in patients with an intermediate (5.0–6.9 mm SA) LLN with at least one malignant feature. LLNDs should be performed according to a minimally-invasive, nerve-sparing technique. If preoperative MRI shows the lateral extension of the tumour into the nerve plexus or an exenteration is required, the patient will still be included to analyse oncological and functional outcomes; however, the patient is not included in the main analyses (see sample size section). If in a shared decision-making process, the patients decide not to undergo an LLND, they remain in the study and are followed for collection of oncological outcomes. In case of a complete clinical response of the tumour and shrinkage of the LLN(s), a patient may enter a watch-and-wait protocol; in these cases, patients should be closely monitored and an LLND may be omitted.

The surgical technique for LLNS has been previously described in detail.^24^ The procedure involves the excision of all lateral lymphatic tissues within the internal iliac and obturator compartment along three anatomical planes: (1) the medial plane defined by the ureter and pelvic plexus (ureterohypogastric fascia), (2) the lateral plane bounded by the psoas and internal obturator muscle and (3) the dorsal plane defined by the internal iliac vessels and the sciatic nerve. It is important to note that the lymphatic tissue along the external iliac arteria is not dissected during this procedure.

### Evaluation of the resection specimen by trained pathologists and grossing assistants

To ensure adequate assessment of resection specimens, pathologists in each participating centre received a protocol (online supplemental file D) to ensure that LLND specimens are adequately and uniformly examined. This protocol was developed by expert pathologists from the LaNoReC study team with experience regarding the assessment of LLND specimens.

### Follow-up and questionnaires

Patients undergo standard oncological follow-up for at least 36 months. At 24 months, a pelvic MRI is advised to evaluate early LLR (see table 1). This MRI will also be reviewed by the participating radiologist. The EORTC-C30, EORTC-CR29, EQ-5D and LARS questionnaires are distributed before surgery and at 6 months, 12 months and 36 months after surgery (table 1). The LARS questionnaire will not be distributed if a non-restorative procedure is performed or if the patient has a permanent or temporary stoma. Surgeons are requested to ask specific questions regarding the evaluation of urinary and sexual dysfunction during follow-up appointments at 6 months and 12 months postoperatively (online supplemental file E).

**Table 1:** Study schedule

Study schedule	Before (C)RT	After (C)RT	6 month-PO	12 month-PO	24 month-PO	36 month-PO
**Questionnaires**	X		X	X		X
**Pelvic MRI**	X	X*			X*	

* Not required.

(C)RT(chemo)radiotherapyPOpostoperative

### Statistics

Analyses will be conducted in Statistical Package for the Social Sciences (SPSS, Chicago, Illinois). LR and LLR will be compared with a percentage of 13% obtained from the two historic cohorts (Consortium and Snapshot) using a one-sample z-test. Baseline characteristics will be described using numbers and proportions for categorical variables; for continuous data, means with 95% CIs or medians with IQR will be used according to the distribution of the data. Clinical data will be described including the type of neoadjuvant treatment, resection margin, height of the tumour, tumour stage (cT/cN stage) and threatened mesorectal fascia (≤1 mm distance). For functional outcomes, descriptive statistics will be used, while quality of life will be analysed using repeated measure analyses. To compare subgroups within the LaNoReC study, χ^2^ tests and Fisher’s exact tests will be performed for categorical variables. Differences in continuous variables will be analysed using student’s t-tests or Mann-Whitney U tests, according to the distribution of the data. A p value of ≤0.05 will be considered statistically significant.

### Patient and public involvement

The Dutch society for colorectal cancer patients (Stichting Darmkanker) were involved in the creation and wording of the patient information folder. Functional and quality of life outcomes in this study are based on comments from patients, an area not often researched after LLND surgery.

### Data management

Data will be prospectively collected and stored in a Castor EDC database (Castor Electronic Data Capture, 2019, online, available at: https://castoredc.com) managed by the central researchers. Specialists in the participating local centres will record data for their included patients. During data entry, they will be able to view other data regarding patients from their own institute, enabling correlation between, for example, radiology slides and radiation fields. Specialists from one centre cannot access the data of patients from other centres.

### Sample size

The sample size is based on a historical control group with an average 3-year LLR rate of 13% (11% Snapshot and 16% Consortium) in low LARC with LLNs ≥7.0 mm after (C)RT+TME (without LLND), which is the current standard treatment in Europe.^5 6 14^ It is hypothesised that (C)RT+TME+LLND can reduce this rate to 6%, as found by the Consortium study. A one-group χ^2^ test with a 5% two-sided significance level will have 90% power to detect the difference between the null hypothesis proportion of 0.13 and the alternative proportion of 0.06 when the sample size is 190. If correcting for a drop-out rate of 5%, 200 patients are needed. Included patients in need of more extensive surgery (ie, pelvic exenteration or presumed inability to spare the hypogastric nerves on MRI) will be registered and analysed as a separate cohort since these more extensive tumours have higher LLR rates and cannot undergo nerve-sparing LLND.

Annually, approximately 882 patients present with low LARC and are treated with neoadjuvant therapy in the Netherlands.^14^ Considering 122 (14%) of these patients have LLNs ≥7.0 mm, a minimum 2.5-year inclusion period is needed to achieve the estimated sample size (expecting 60% of eligible patients to be enrolled).^14^ As some of the eligible patients will undergo open surgery, it is expected that the inclusion period may require an extension. An interim analysis will be carried out after the inclusion of the first 75 participants. The goal of this interim analysis is to evaluate early indicators of effectiveness concerning oncological outcomes, particularly LLR rate, and to evaluate safety by examining short-term surgical complications (within 90 days) and early functional outcomes (urogenital dysfunction within 3 months). The results will help improve the preoperative information provided to patients. If the interim analysis reveals a high LLR rate that suggests that the hypothesis may not be achievable or significant morbidity within 3 months of surgery, the study team will consider to prompt the study ahead of time.

Additionally, patients with rectal cancer and an LLN of 5.0–6.9 mm SA with at least one malignant feature will be investigated. For this analysis, a sample size calculation is impossible due to limited data. Therefore, all eligible patients in this group will be collected during the study period.

### Ethics and dissemination

The medical ethics board of the VU Medical Centre, the Netherlands, centrally approved the study on 23 November 2022 (reference 2021.0524). All participating centres need to obtain local approval of the study protocol and all patients will provide written informed consent before participation. Results obtained from the data collected in this study will be published in peer-reviewed medical journals.

## Discussion

The LaNoReC study is the first study that will evaluate oncological and functional outcomes of optimal standardised care for rectal cancer patients with enlarged LLNs after dedicated training of the whole multidisciplinary team. An earlier study showed that this training leads to significant improvement in reporting by radiologists,^27^ consistent delineation by radiation oncologists, nerve-sparing minimally invasive LLND by surgeons and assessment of the pathological specimen with attention for LLNs. Considering the high risk of LLR for those with enlarged LLNs when treated with neoadjuvant therapy and TME alone, the LaNoReC study will evaluate whether these refined treatment strategies including adequate radiotherapy and LLND surgery will result in a reduction of 3-year LLR rate from 13% to 6%, corresponding to 15 adequately treated patients to prevent one LLR.

The proposed study design is a prospective registration study with detailed data collection and outcome analysis, in which training has been applied to the various aspects of the diagnostic process and subsequent treatment. This study design, rather than a randomised controlled trial, was chosen because an earlier attempt in 2016 by Wei *et al* to investigate the additional value of LLND after neoadjuvant CRT in a randomised setting was terminated early due to low enrolment.^28^ Moreover, it would be hard to define a control group, because current practice comprises very heterogeneous treatment strategies. In addition, it was chosen not to impose actions (such as mandatory CRT without a boost), because due to new evidence and training of the multidisciplinary team in participating centres, the control intervention does not exist anymore. This might be overcome by cluster randomised trials, but there is a high risk of adapting routine practice towards the study intervention. Moreover, there is a lack of equipoise considering the optimised treatment versus routine practice, and one might even state that it is unethical to withhold patients the most optimal treatment. As a result, the prospective registration nature of this study was found to be most appropriate.

The inclusion criteria for the LaNoReC study are based on the Snapshot and the Consortium study, in which the combination of (C)RT and TME was insufficient for adequate treatment of patients with enlarged LLNs.^5 14^ Besides short-axis size, the recurrence rates seem to be also influenced by other radiological criteria, such as the presence of malignant features which increased the LLR rate in intermediate LLNs to 8% (vs 2% in absence of malignant features). In enlarged nodes, the presence of two or more malignant features was associated with an increased LLR rate of 17% after 4 years, while no LLR developed if such features were absent. Since the influence of malignant features has only been described in small subgroups and should be interpreted with caution, the inclusion criteria and LLND recommendations for the LaNoReC study are based on short-axis size only for enlarged nodes and intermediate nodes specifically combined with the presence of malignant features. By including this intermediate node group, the LaNoReC study aims to dissolve the current lack of knowledge regarding treatment options and oncological outcomes in those patients. After thorough evaluation during multidisciplinary meetings and a shared decision-making process with the patient, an LLND can be considered to lower the LLR rate.

The inclusion criteria described in the previous paragraph should be based on primary MRI, as the Consortium study is the only study which found that sufficient downstaging on restaging MRI (shrinkage of iliac and obturator LLNs to ≤4 mm and ≤6 mm, respectively) could avoid LLND.^5^ The Snapshot study, however, showed that downsizing of LLNs below the Consortium cut-offs still resulted in an unacceptably high LLR rate of up to 15%.^14^ The inclusion criteria for the LaNoReC study are based on the results of the Snapshot study because of the high validity of these findings due to the training of radiologists. Exempted from this are those with a complete clinical response for which a watch-and-wait trajectory is increasingly applied. Although these patients were not included in the Snapshot study, and therefore restaging results are not applicable to this group, we believe an LLND can be omitted in these cases and close surveillance is justified.

In this study, patients can be treated with different neoadjuvant schedules, provided it contains radiotherapy. This allows the multidisciplinary team to choose a treatment strategy based on tumour features, such as extramural venous invasion or tumour deposits. Although an increased LR rate for patients treated with the RAPIDO schedule has been reported, these patients will still be included together with the other neoadjuvant schemes to increase external validity and optimal accrual of the study, thereby avoiding allocation bias related to disease characteristics that will guide decision-making.^26 29^

There are numerous strengths to this study. First, the Dutch participating centres have participated in validated training sessions by experts from the LaNoReC study team, these training sessions will be organised again for interested international centres, which are only allowed to join when the whole multidisciplinary team has sufficed the training criteria. Working internationally increases the external validity of the study and allows for a wide range of eligible patients to be included. Most importantly, this study depends on and strengthens multidisciplinary collaboration and allows for the direct implementation of accurate diagnostics and treatment. This teamwork will increase awareness in all involved specialties and encourage active communication regarding LLNs during multidisciplinary meetings, which will increase individual accuracy during diagnostics and treatment.

Currently, there is no other prospective study investigating the outcomes of LLND in patients with enlarged LLNs. Therefore, the LaNoReC study will provide new and insightful information regarding this newly developed treatment strategy. While a decrease in LLR is essential for the accordance and universal establishment of multidisciplinary treatment, quality of life and morbidity outcomes will also provide novel insights into this treatment in Western patients.

## supplementary material

10.1136/bmjopen-2023-083225 online supplemental file 1

10.1136/bmjopen-2023-083225 online supplemental file 2

10.1136/bmjopen-2023-083225 online supplemental file 3

10.1136/bmjopen-2023-083225 online supplemental file 4

10.1136/bmjopen-2023-083225 online supplemental file 5

## Data Availability

Data are available upon reasonable request.
