# Supplementary material for: Optimised treatment of patients with enlarged lateral lymph nodes in rectal cancer: protocol of an international, multicentre, prospective registration study after extensive multidisciplinary training (LaNoReC)
Source: BMJ Open. 2024 Oct 16;14(10):e083225. doi: 10.1136/bmjopen-2023-083225 (PMC11487837; doi:10.1136/bmjopen-2023-083225)
Supplement: online supplemental file 1 [file bmjopen-14-10-s001.pdf]

Supplementary file A. Patient informed consent forms

Subject information for participation in a medical-scientific study

LaNoReC study

Official title: Lateral Nodal Recurrence in Rectal Cancer

Introduction

Dear Sir or Madam,

You are being asked to take part in a medical-scientific study. You are receiving this letter because you have rectal cancer. You also have at least one enlarged lymph node outside the rectum, we call these lateral lymph nodes. Participation in this study is voluntary. In order to participate your written consent is required. The Medical Ethics Committee of the Amsterdam UMC has decided that this study is exempt from the Dutch Research Involving Human Subjects Act (WMO).

Before you decide whether you want to take part in this study, you will be given an explanation about what the study involves. Please take your time to read this information and ask the investigator if you have any questions. You can also discuss it with your partner, friends or family.

1. Purpose of the study

The main purpose of the study is register all patients with rectal cancer and enlarged lateral lymph nodes (outside the rectum). In the past, these lateral lymph nodes were not treated adequately. New evidence, more awareness and training of clinicians have resulted in better staging and improved treatment. The LaNoReC study aims to follow patients to investigate whether the developments have improved outcomes. Therefore, we would like to register the details of your treatment.

2. Background information

You have been diagnosed with rectal cancer. At least one enlarged lymph node has also been identified on the initial MRI outside the rectum, also known as ‘lateral’ lymph nodes. When these lymph nodes are enlarged, they may contain tumour cells, which may result in recurrence of the tumour when not adequately treated. Since the treatment of these lateral lymph nodes has improved in recent years, this study aims to evaluate whether the chance of recurrence has decreased.

For this study, radiologists have been trained to notice and adequately assess these lymph nodes, to carefully select patients with enlarged lateral lymph nodes. Radiation oncologists have been trained to delineate these lateral lymph nodes, which aims to shrink the tumour and lateral lymph nodes. You may also receive chemotherapy. After this, a surgeon will discuss the option to remove the lateral lymph nodes with an operation. The operation to

remove these lymph nodes already takes place in other countries; however, others have little experience with this technique. Mainly because until recently, the role of lateral lymph nodes in recurrence development was not known. Furthermore, the operation is more complex if the lateral lymph nodes are also removed, due to many nerves in the surgical area which need to be spared. With this study we want to ensure that, if you require this surgical procedure, it is done by an experienced surgeon following an established nerve-sparing technique. This combination of adequate assessment of lateral lymph nodes, irradiation and the operation is known to decrease the risk of recurrence.

If you decide not to participate in this study, this will not affect your treatment, as the above mentioned trainings have been implemented in your hospital and are considered standard of care.

3. What participation involves

This study collects information about your clinical course, imaging, radiotherapy, surgery, hospitalization and oncological follow-up. During the study these data will be collected from your electronic patient file. In addition, four questionnaires will be distributed to you at four moments (before surgery, 6, 12 and 36 months after surgery). These questionnaires provide insights in the disease burden and quality of life. All information will be stored in a secure database without any personal data that would reveal your identity.

4. Possible advantages and disadvantages

It is important that you properly consider the possible advantages and disadvantages before you decide to participate.

If you participate in this study, there are no direct advantages. A disadvantage of participation in this study is the additional time required to complete questionnaires at 4 moments during 3 years

5. If you do not want to participate, or would like to stop participating in the study at any time

You decide for yourself whether you want to participate in the study. Participation is voluntary.

If you do participate, you can always change your mind and withdraw yourself, even during the study. You do not have to state why you are withdrawing. However, you should immediately inform the investigator. The data obtained thus far will be used for the study.

6. Use and storage of your data

For this study, your personal data will be collected, used and stored. It involves information such as your name, year of birth, data about your health, and data about the therapy you receive. We ask your consent for the use of your data.

Confidentiality of your data
To protect your privacy, your data will receive a code. Your name and other information that could directly identify you are omitted. This information can only identify you with the key. The key to the code will be stored securely in the local research facility. The data that is sent to the sponsor [and any other parties involved] only contain a code, but not your name or other data that can identify you. In reports or publications about the study, the data will also not be identifiable.

Retention period of data and use for other studies
Your data may still be of interest after the end of this study for other clinical research in the area of illness. For this your data will be stored for 15 years at the study site and 15 years at the sponsor. If you choose to participate in the study, you consent with the retention period of data and the use for other clinical research.

More information about your rights concerning the processing of data
For general information about your rights concerning the processing of your personal data, please consult the website of the Dutch Data Protection Authority (https://autoriteitpersoonsgegevens.nl/). If you have any questions about your rights, please contact the person responsible for the processing of your personal data, which you can find in the contact details section.

If you have any questions or complaints regarding the processing of your personal information, we recommend that you contact the study site. You can also contact the Data Protection Officer for the Amsterdam UMC (privacy@amsterdamumc.nl) or the [insert other institution name refer to contact details] or the Dutch Data Protection Authority.

7. Compensation for participation

You will not receive compensation for participation in this study.

8. Do you have any questions?

If you have any questions, please contact the investigational team.

If you have any complaints about the study, you can discuss this with the investigator or your treating physician. If you would rather not do that, you can contact the complaints committee at your hospital or that complaints officer of the Amsterdam UMC, location VUmc through privacy@vumc.nl.

Contact details for the research team in Amsterdam UMC

Coordinating investigator: E. van Geffen, lanorec@amsterdamumc.nl, +3120-4444444
Principal investigator: Dr. M. Kusters, +3120-4444444

Amsterdam UMC, location VUmc

Surgery department
De Boelenlaan 1117
1081 HV Amsterdam
The Netherlands

Appendix: consent form

LaNoReC study

- I have read the information letter. I was also able to ask questions. My questions have been answered sufficiently. I have had enough time to decide whether or not to participate.
- I understand that participation is voluntary. I also know that I may decide at any time to not participate or to stop participating in the study. Without having to provide any reason.
- I give consent to collect and use my data for answering the research question in this study
- I give consent that my data is sent and stored at Amsterdam UMC for 15 years

I voluntarily agree to participate in this study.

Name of subject: ……………………………………………………………………………..
Signature: Date : __ / __ / __

I certify that I have fully informed this subject about the said study.

If information becomes known during the study that could influence the consent of the subject, I will inform him/her of this on time.

Name of investigator (or his/her representative):…………………………………………
Signature: Date: __ / __ / __

---

* Tick the appropriate box

The subject will receive a complete information letter, together with a signed version of the informed consent form.
