# Supplementary material for: Optimised treatment of patients with enlarged lateral lymph nodes in rectal cancer: protocol of an international, multicentre, prospective registration study after extensive multidisciplinary training (LaNoReC)
Source: BMJ Open. 2024 Oct 16;14(10):e083225. doi: 10.1136/bmjopen-2023-083225 (PMC11487837; doi:10.1136/bmjopen-2023-083225)
Supplement: online supplemental file 2 [file bmjopen-14-10-s002.pdf]

Supplementary file B. MR Imaging Protocol LaNoReC

Hardware

1.5 / 3.0 T

External Phased Array Coil (no endorectal coil)

Patient Preparation

Spasmolytics may be used depending on local protocol. Spasmolytics can be beneficial when significant bowel movement artefacts are visible on the planning images, especially 3T. Use of enema is not routinely recommended.

Sequences and sequence angulation

Imaging should be performed according the ESGAR recommendations: Magnetic resonance imaging for the clinical management of rectal cancer patients: Updated recommendations from the 2016 European Society of Gastrointestinal and Abdominal Radiology (ESGAR) consensus meeting. Eur Radiol (2018) 28(4):1465-1475

2D T2-weighted sequences

- Sagittal, coronal and axial 2D T2-weighted sequence is mandatory for the assessment of tumour height, T-N-stage, MRF involvement and the presence of EMVI
- Axial and coronal T2-weighted sequence should be angulated perpendicular and parallel to the tumour axis for tumours in the middle part of the rectum
- For low rectal tumours, angulation depends on the extent of the tumour and may be performed perpendicular and parallel to either the tumour axis or the anal canal, or even both (4 series)
- Slice thickness: £ 3mm
- FOV: cranial border: upper side L5 / caudal border: beyond anal canal

Use of DWI is not obligatory for primary staging but is recommended for restaging (specifically for assessment of the T-stage) after CRT. B800-1000

No 3D T2-weighted and fat-suppressed sequences and no T1-weighted sequence after administration of intravenous contrast medium.

T2 sequence: based on the following reference: Horvat, N., Carlos Tavares Rocha, C., Clemente Oliveira, B., Petkovska, I., & Gollub, M. J. (2019). MRI of rectal cancer: Tumor staging, imaging techniques, and management. Radiographics, 39(2), 367-387.

MR staging

MR Images are judged on:

- Position of tumour
    - Distance to anorectal junction
    - Circumferential extent
    - Distance to the cranial border internal sphincter
    - Location (anterior, right lateral, posterior, left lateral)
- Length of tumour
- T-status
    - T0: no evidence of primary tumour
    - Tis: carcinoma in situ: intra-epithelial or invasion of lamina propria
    - T1: tumour invades submucosa
    - T2: tumour invades muscularis propria
    - T3a: tumour invades beyond muscularis <1mm
    - T3b: tumour invades beyond muscularis 1-5mm
    - T3c: tumour invades beyond muscularis 5-15mm
    - T3d: tumour invades beyond muscularis >15mm
    - T4a: tumour perforates visceral peritoneum
    - T4b: tumour invades directly into other organs or structures
- Distance of the tumor to mesorectal fascia (in mm)
    - Other lesion (EMVI, tumor deposits, lymph nodes) situated within 1mm of the MRF
- EMVI: extramural vascular invasion: including size in mm
    - Negative:
        - Grade 0: pattern of tumor extension through the muscle coat is not nodular, and there is no vessels adjacent to areas of tumor penetration
        - Grade 1: minimal extramural stranding/nodular extension, but not in the vicinity of any vascular structure
        - Grade 2: stranding demonstrated in the vicinity of extramural vessels, but these vessels are of normal caliber, and there is no definitive tumor signal within the vessel
    - Positive:
        - Grade 3: intermediate signal intensity apparent within vessels, although the contour and caliber of these vessels is only slightly expanded
        - Grade 4: obvious irregular vessel contour or nodular expansion of vessel by definitive tumor signal

- N-status
    - Malignant criteria:
        - Irregular borders
        - Round shape
        - Heterogeneous signal intensity
    - N0: no suspicious mesorectal lymph nodes and no LLNs or LLNs < 7.0mm
    - N status lateral lymph nodes:
        - Number of visible lateral lymph nodes
        - Description of compartment of visible nodes (internal iliac, obturator or external iliac compartment)
        - Short-axis dimension in mm (including cortex) of the LLN(s) on an axial/sagittal or coronal image.
        - Malignant criteria:
            - Irregular borders
            - Round shape
            - Heterogeneous signal intensity
            - Loss of fatty hilum
        - Diffusion restriction
- Presence of tumor deposits
- M-status (if possible)
