# Supplementary material for: Optimised treatment of patients with enlarged lateral lymph nodes in rectal cancer: protocol of an international, multicentre, prospective registration study after extensive multidisciplinary training (LaNoReC)
Source: BMJ Open. 2024 Oct 16;14(10):e083225. doi: 10.1136/bmjopen-2023-083225 (PMC11487837; doi:10.1136/bmjopen-2023-083225)

Supplementary file C: Delineation guideline rectal cancer: LPRGE consensus 2023

F. Peters and M. Intven, translated into English by T. C. Sluckin and E.G.M. van Geffen

Adaptation of the delineation guidelines by Vincenzo Valentini et al., International consensus guidelines on Clinical Target Volume delineation in rectal cancer, Radiotherapy and Oncology 120 (2016) 195–201. The instructions for the mesorectum are based on the STAR-TReC study protocol.

General information
The mesorectal, anal- and ischio-rectal fossa (IRF) areas and the lymph node compartments are delineated separately as different PTV margins can be used for these regions. These varying PTV margins result from large internal movements of the mesorectum (due to rectal and bladder filling), the anus and the IRF with respect to the lymph node compartments.

Target areas

1. Primary tumour
- Tumor margins: In the case of a threatened MRF, at least a 5mm circumferential margin should be added to the tumour adjusted for 'not involved' organs. In the case of expansion of the tumour above the mesorectal level, always at least 1 cm, following the intestine cranially and 1cm circumferentially is added around the tumour and adjusted for surrounding 'not involved' organs

2. Mesorectum (mesorectum (M))
Indication: always
- Cranial: S2-3
- Caudal: at least 2cm caudal of the primary tumour, also when the tumour expands into the anus. In this case, delineation of the internal sphincter is required, see point 3
- Ventral: mesorectal fascia, when the mesorectal fascia disappears, the ventral border is the anterior rectal wall and above the level of the rectum, an extension of the same anterior border
- Dorsal: sacrum, pelvic wall (the pre-sacral lymph node volume in the mesorectal target area at this level are included)
- Lateral: mesorectal fascia
- Medial: not applicable

3. Pre-sacral lymph nodes (pelvic presacral nodes (PN))
Indication: always
- General: always a minimal margin of 5 mm around pathological lymph nodes, adjusted to 'not involved' organs
- Cranial: S1-S2, always at least 5 mm above the most cranial pathological lymph node. With non-locally advanced tumours <10 cm from the anus and N0 status, the cranial border can be lowered to S2-S3 in accordance with Nijkamp et al. (*IJROBP 2011; 80(1) 103-110*)
- Caudal: cranial boarder of the mesorectum
- Ventral: a minimal margin of 1cm is required ventral of the sacrum, perpendicular to the sacrum and 7mm around the superior rectal artery, yet not beyond the ventral boarder of the delineated internal iliac lymph nodes (see point 4)
- Dorsal: sacrum, pelvic wall muscles, do not include neuroforamina
- Lateral: the iliac lymph node area (IIN)
- Medial: not applicable

4. Posterior lateral lymph nodes (before: internal iliac lymph nodes/internal iliac nodes (IIN))

Indication: always

- General: always a minimum margin of 5mm around pathological lymph nodes, adapted to 'not involved' organs
- Cranial: the caudal bifurcation of the communal iliac vein, maximal S1-S2, yet always at least 5mm above the most cranial pathological lymph node. With non-locally advanced tumours <10 cm from the anus and N0 status, the cranial boarder of the CTV can be lowered to S2-S3 in accordance with Nijkamp et al. (*IJROBP 2011; 80(1) 103-110*)
- Caudal: level where the internal iliac artery exits the pelvic region and the sacrotuberal ligament becomes apparent (see figure 1)
- Ventral: 7mm ventral of the main branch of the internal iliac vein and artery.
- Dorsal: pre-sacral lymph nodes, mesorectum, pelvic wall
- Lateral: pelvic wall. Dorsal of the acetabulum, the medial border of the musculus obturatorius internus is the lateral border (see figure 2)
- Medial: mesorectum or 7mm around vessels

5. <u>Anterior lateral lymph nodes (before: obturator compartment/lateral lymph nodes (LLN)</u>
   Indication: when the tumour is situated under the peritoneal reflection AND one of the following criteria: anal sphincter invasion, N2 status, cT4 or pathological internal iliac lymph nodes

- General: always a minimum margin of 5mm around pathological lymph nodes, adapted to 'not involved' organs
- Cranial: to the highest level where the obturator muscle is visible
- Caudal: level were the levator ani muscle enters the pelvic wall (best visible on coronal plane: figure 3)
- Ventral: halfway the obturatorius internus muscle (best visible on axial plane)
- Dorsal: ventral boarder of the posterior lymph node compartment, pelvic wall
- Lateral: pelvic wall. Dorsal of the acetabulum, the medial border of the musculus obturatorius internus is the lateral border (figure 2)
- Medial: 7mm around large vessels, unless mesorectum/sigmoid or peritoneum are visible; then use them as the medial border. In the caudal part of the level where the internal iliac artery enters the pelvis, the mesorectum forms the medial boundary. Avoid the uterus, vagina, seminal vesicles, parametrica, and neurovascular bundle (figure 2)

6. <u>Sphincter complex (SC)</u>
   Indication: if the tumour or mesorectal caudal extension (see above) expands into the anus

- Delineation: sphincter complex up to 2 cm below the tumour
  i) If the tumour does not infiltrate the anal muscles: delineate only the internal sphincter
  ii) If the tumour infiltrates the internal sphincter: delineate the internal and external sphincter
  iii) If the tumour infiltrates the external sphincter: delineate the internal and external sphincter and the ischiorectal fossa area (see point 7)

7. <u>Ischiorectal fossa (ischio-rectal fossa (IRF))</u>
   Indication: infiltration of the external sphincter or the ischio-rectal fossa

- Delineate 1cm around the GTV and sphincter

8. <u>Abdominal pre-sacral lymph nodes (abdominal presacral nodes (Abd PS))</u>
   Indication: consider including this area if tumour-positive lymph nodes are located here

- Caudal: promontorium, to join the presacral lymph node (PN) area
- Other borders: at least 5 mm around affected lymph nodes

9. <u>Inguinal lymph nodes (inguinal nodes (IN))</u>
Indication: consider including this area in case pathological lymph nodes are located here

- General: always a minimum margin of a 5mm around pathological lymph nodes, adapted to 'not involved' organs
- Cranial: where the circumflex vein (deep part) and external iliac artery cross each other. If this is poorly visible on CT: between the acetabulum roof and the superior arm of the pubis bone
- Caudal: the insertion of the great saphena vein in the femoral vein
- Ventral: at least 20mm margin around inguinal vessels including visible lymph nodes or a lymphocele
- Dorsal: a triangle formed by the iliopsoas muscle, pectineus muscle and long abductor muscle
- Lateral: medial boarder of the sartorius or iliopsoas muscle
- Medial: 10-20mm margin around the femoral vessels, including visible lymph nodes or a lymphocele

	Mesorectum	Pre-sacral LN	Posterior lateral LN	Anterior lateral LN	Sphincter complex	Ischiorectal fossa	Abdominal pre-sacral LN	Inguinal LN
For all indications	✓	✓	✓					
Below peritoneal reflection AND one of the following: anal sphincter invasion, N2, cT4, or extramesorectal lymph node	✓	✓	✓	✓				
Abdominal pre-sacral pathological node	✓	✓	✓	✓			✓	
Inguinal pathological node	✓	✓	✓	✓				✓
Infiltration internal sphincter	✓	✓	✓		✓			
Infiltration external sphincter of ischiorectal fossa	✓	✓	✓		✓	✓		

Figure 1.

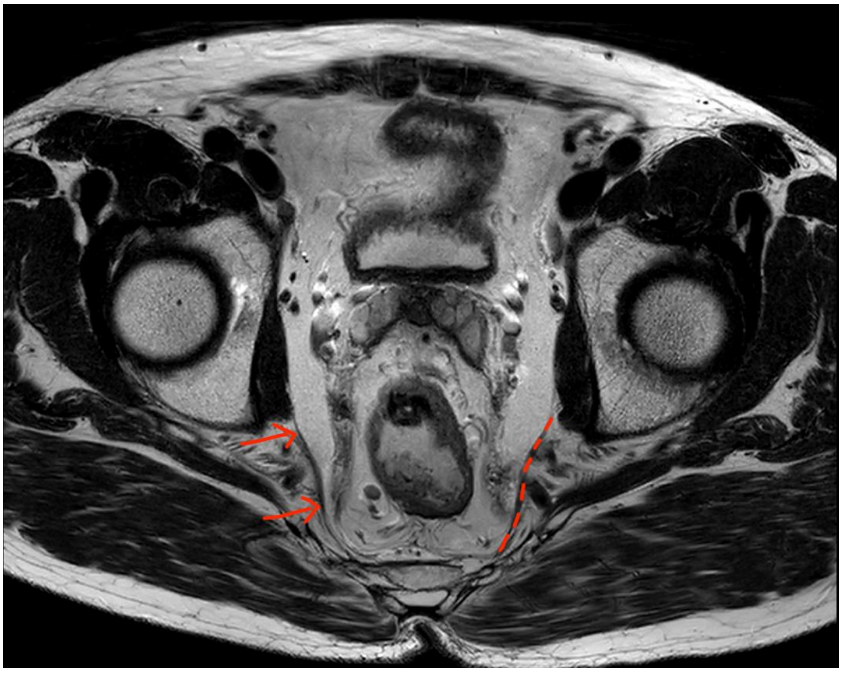

Figure 2.

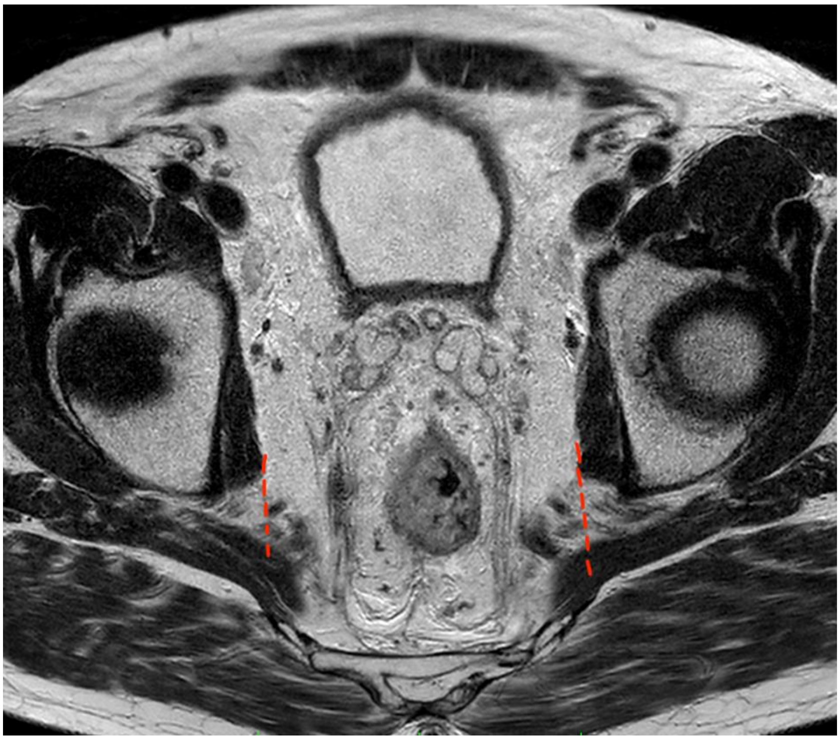

Figure 3.

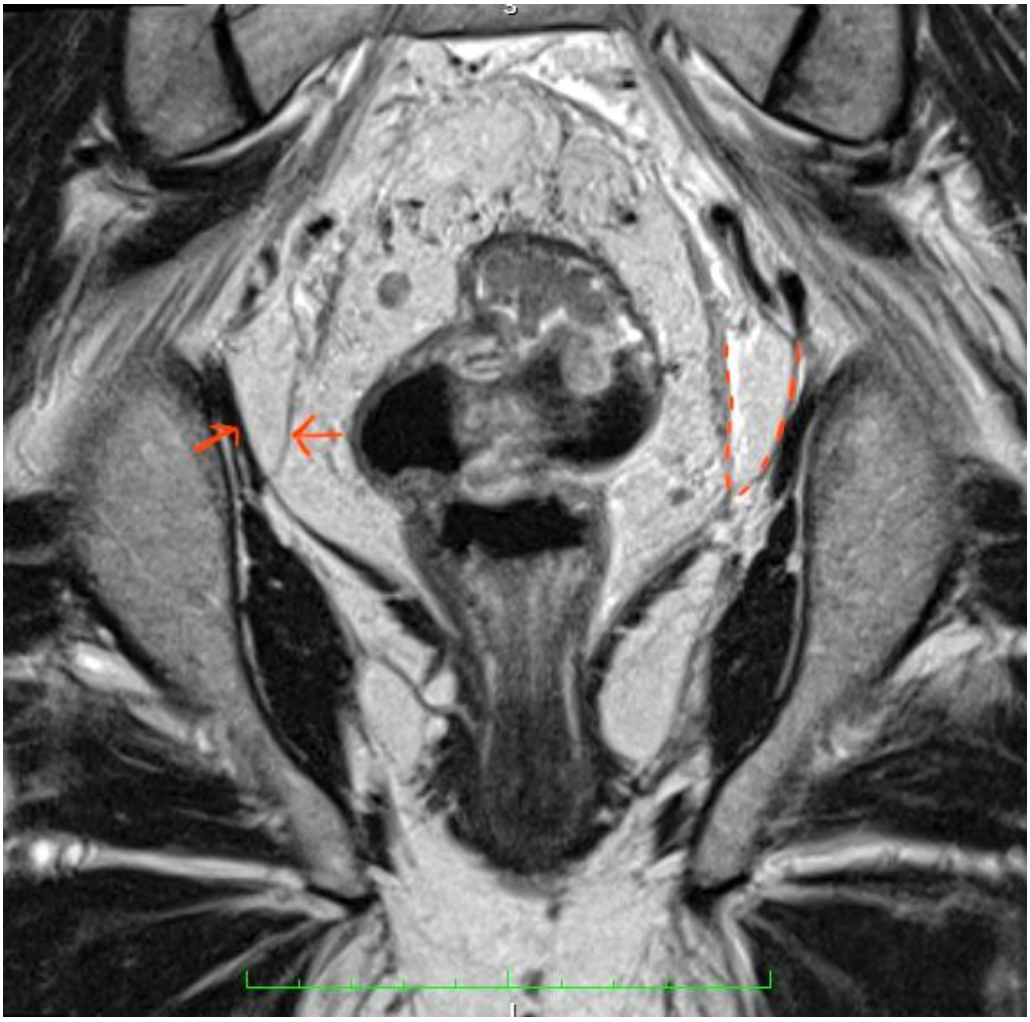

Supplement: online supplemental file 3 [file bmjopen-14-10-s003.pdf]
