# Supplementary material for: Optimised treatment of patients with enlarged lateral lymph nodes in rectal cancer: protocol of an international, multicentre, prospective registration study after extensive multidisciplinary training (LaNoReC)
Source: BMJ Open. 2024 Oct 16;14(10):e083225. doi: 10.1136/bmjopen-2023-083225 (PMC11487837; doi:10.1136/bmjopen-2023-083225)
Supplement: online supplemental file 4 [file bmjopen-14-10-s004.pdf]

Supplementary file D. Pathology Protocol

This specimen originates from a patient included in the LaNoReC study and contains tissue from a lateral lymph node dissection. In this study oncological outcomes in patients with rectal carcinoma with enlarged or suspicious lateral lymph nodes are investigated. It is of utmost importance to examine **all** lymph nodes in this fatty tissue. Therefore the specimen should be handled carefully to maximize the lymph node yield.

Lymph nodes should be cut out and submitted for microscopic examination according to the following standard procedure:

1. Measure the specimen in three dimensions. If a macroscopic picture with a ruler next to the specimen has not been taken in the operating room and uploaded in the electronic patient file, please take a picture of the specimen with a ruler next to it.
2. It is preferred not to use fat dissolving chemicals, since this might influence the yield of the lymph nodes. If this cannot be avoided, please note this in the report.
3. Identify all lymph nodes by careful inspection, palpation and finely dissecting the tissue. Submit every lymph node using the following guidelines:
    - Small lymph nodes (≤0.3 cm) should not be bisected or sliced and can be submitted with a maximum of 4 per cassette.
    - Lymph nodes between 0.3 and 0.6 cm should be bisected along the median plane and can be submitted with a maximum of 2 lymph nodes per cassette (in which case one lymph node should be inked before bisecting)
    - Larger lymph nodes should be sectioned into slices of 0.3 cm and submitted completely in 1 or more cassettes. If it concerns a very fatty lymph node (>2 cm) it is sufficient to submit the slices alternately.
    - Solid areas which are not obvious lymph nodes, should be treated in a similar way because these often contain small nodes.

Exceptions:

- Submitting one section of an obvious macroscopically involved node is sufficient. In this case the largest macroscopically involved lymph node should be measured. Where extracapsular extension is apparent or suspected, please submit this area.
- Sometimes several lymph nodes form a firm pack together. Cut this tissue into thin slices (0.3 cm) and try to determine the number of lymph nodes. In this case one section per lymph node is sufficient.
