# Supplementary material for: Optimised treatment of patients with enlarged lateral lymph nodes in rectal cancer: protocol of an international, multicentre, prospective registration study after extensive multidisciplinary training (LaNoReC)
Source: BMJ Open. 2024 Oct 16;14(10):e083225. doi: 10.1136/bmjopen-2023-083225 (PMC11487837; doi:10.1136/bmjopen-2023-083225)
Supplement: online supplemental file 5 [file bmjopen-14-10-s005.pdf]

Supplementary file E. CRFs functional outcome

20.13.2	***If 'Does the patient currently have a urinary catheter at this moment (permanent or intermittent)?' is not equal to 'Yes' answer this question:***<br>Which phrase best describes the current situation: multiple answers possible	☐ No urinary problems<br>☐ Subjective problems with fully emptying the bladder<br>☐ Frequent urination<br>☐ Difficulty in delaying urination
20.13.2.1	***If 'Which phrase best describes the current situation: multiple answers possible' is not equal to 'No urinary problems' answer this question:***<br>How often are urinary problem present?	○ <20% of times<br>○ 20-50% of times<br>○ 50-75% of times<br>○ >75% of times<br>○ Almost always
20.14	***If 'Gender' is equal to 'Male' answer this question:***<br>Which phrase best describes the current situation: multiple answers possible	☐ Not sexually active<br>☐ Current erectile dysfunction<br>☐ Current ejaculation dysfunction<br>☐ No problems with either erection or ejaculation
20.14.1	***If 'Which phrase best describes the current situation: multiple answers possible' is not equal to 'No problems with either erection or ejaculation' answer this question:***<br>How often are sexual problems present?	○ Not sexually active<br>○ <20% of the time<br>○ 20-50% of the time<br>○ 50-75% of the time<br>○ >75% of the time<br>○ (nearly) always
20.14.2	***If 'Which phrase best describes the current situation: multiple answers possible' is equal to 'Current erectile dysfunction' answer this question:***<br>If erectile dysfunction is present, is this only since the operation or also before?	○ Present only since the operation<br>○ Present also before the operation
20.14.3	***If 'Which phrase best describes the current situation: multiple answers possible' is equal to 'Current erectile dysfunction' answer this question:***<br>If ejaculation disfunction is present, is this only since the operation or also before?	○ Present only since the operation<br>○ Present also before the operation
20.15	***If 'Gender' is equal to 'Female' answer this question:***<br>Which phrase best describes the current situation: multiple answers possible	☐ Not sexually active<br>☐ Current pain during penetration<br>☐ Current lubrication ('wetness') problems<br>☐ No problems with either pain during penetration or lubrication
20.15.1	***If 'Which phrase best describes the current situation: multiple answers possible' is not equal to 'No problems with either pain during penetration or lubrication' answer this question:***<br>How often are sexual problems present?	○ Not sexually active<br>○ <20% of the time<br>○ 20-50% of the time<br>○ 50-75% of the time<br>○ >75% of the time<br>○ (nearly) always
20.15.2	***If 'Which phrase best describes the current situation: multiple answers possible' is equal to 'Current pain during penetration' answer this question:***<br>If pain during penetration is present, is this only since the operation or also before?	○ Present only since the operation<br>○ Present also before the operation
20.15.3	***If 'Which phrase best describes the current situation: multiple answers possible' is equal to 'Current lubrication ('wetness') problems' answer this question:***<br>If lubrication problems are present, is this only since the operation or also before?	○ Present only since the operation<br>○ Present also before the operation
