# Supplementary material for: Optimised treatment of patients with enlarged lateral lymph nodes in rectal cancer: protocol of an international, multicentre, prospective registration study after extensive multidisciplinary training (LaNoReC)
Source: BMJ Open. 2024 Oct 16;14(10):e083225. doi: 10.1136/bmjopen-2023-083225 (PMC11487837; doi:10.1136/bmjopen-2023-083225)
Supplement: Supplementary file 6 [file bmjopen-2023-083225.reviewer_comments.pdf]

PEER REVIEW HISTORY

BMJ Open publishes all reviews undertaken for accepted manuscripts. Reviewers are asked to complete a checklist review form and are provided with free text boxes to elaborate on their assessment. These free text comments are reproduced below.

ARTICLE DETAILS

Title (Provisional)

Optimized treatment of patients with enlarged Lateral lymph Nodes in Rectal Cancer: protocol of an international, multicentre, prospective registration study after extensive multi-disciplinary training (LaNoReC)

Authors

van Geffen, Eline G. M.; Sluckin, Tania C.; Hazen, Sanne-Marije J. A.; Horsthuis, Karin; Intven, Martijn; van Dieren, Susan; Beets, Geerard; Lange, Marilyne M.; Taggart, Melissa W.; Beets-Tan, Regina G. H.; Marijnen, Corrie A. M.; Konishi, Tsuyoshi; Tanis, Pieter J; Kusters, Miranda

---

VERSION 1 - REVIEW

**Reviewer**	**1**
**Name**	**Agger, Erik**
**Affiliation**	**Lund University**
**Date**	**02-Mar-2024**
**COI**	**None**

Dear Dr Kusters and colleagues,

Thank you for giving me chance to review this study protocol.

The Dutch group proposes a prospective study describing the outcome of a standardized treatment protocol of suspected lateral lymph node metastases in rectal cancer.

The reseach question is clinically relevant and adresses an identified source of local recurrence in rectal cancer. Treatment strategies are debated however there is growing consensus that surgical treatment of suspected lateral lymph node metastases needs to be further explored even in the era of advanced neoadjuvant oncological therapy. The lymph nodes in the lateral internal iliac and obturator area are clearly defined which is a methodological strenght.

The expected effect in local recurrence reduction appear reasonable based on previous studies from Europe and Asia and could possibly be shown by the study in its current design.

I have a few comment:

1. In describing the study outcome, both DFS and OS are suggested as outcome measures. Why did you not just go for OS?

One could argue that OS alone would sufficient and a more robust outcome measure especially when introducing lateral lymph node dissection which might increase the number of surgical adverse events.

2. The protocol suggests re-staging MRI to be optional. Why did you not plan to make re-staging MRI mandatory in the protocol?

I understand that the surgical indication will not be affected,

however, systematic re-staging MRI could possibly provide valuable additional information to this otherwise ambitious project.

3. Several neoadjuvant regimes will be used. Do you expect a difference in oncological effect on lateral nodes depending on given CRT or RAPIDO-style therapy? Would it be interesting to perform a pre-defined subgroup analysis of this? Would such a analysis be adequately "powered" in your opinion based on the expected distibution between CRT/RAPIDO?

4. Please define LLND more clearly in the protocol. I think you are going to use en-bloc compartment dissection instead of node-picking? If so please describe.

5. How will patients in which complete lymph node dissection is not done/possible be managed during follow-up and described? Exclusion from study?

6. Which type of surgical resections will be included and in which expected numbers?

Lateral lymph nodes are more common in medium/low rectal cancer. It might be expected that a sizeable proportion will undergo an APR with permanent colonostomy. Could you provide expected numbers and discuss this in relation to your planned follow-up with LARS-questionnaires?

7. You expect a drop-off of 5% and have sized the study cohort thereafter. What do you base 5% on. Would i be reasonable to increse the cohort expection a 10% drop-off?

8. You are planning an interim-analysis. Please clearly define how this will be perfored and which parametres this will include. Please also define under which circumstances the interim analysis could prompt the study to be closed ahead of time.

**Reviewer**	**2**
**Name**	**Ryoo, Seung-Bum**
**Affiliation**	**Seoul National University College of Medicine, Surgery**
**Date**	**13-May-2024**
**COI**	**none**

I have reviewed the manuscript of ‘Optimized treatment of patients with enlarged lateral lymph nodes in rectal cancer: protocol of an international, multicenter, prospective registration study after extensive multi-disciplinary training (LanoRec)’. This manuscript is about a study protocol for prospective registration study. This is a very important study and expected to get the significant results, but there are some points to be considered.

1. Evaluation of surgical procedure and standardization is key to success in the trials for surgical procedures. In your study, you have planned that LLNDs will be performed by a select team of surgeons after completing adequate human cadaver training, intensive proctoring and assessment of procedural videos. Are there any evaluating tools or organized check-list in your protocol to guarantee the performance of well-trained surgeon or standardization of the procedures?

2. LLNDs can improve the oncologic outcomes, but might be harmful to functional outcomes, related to sexual or urinary functions. For secondary outcomes, do you have plan to evaluate specific functional outcomes about those?

VERSION 1 - AUTHOR RESPONSE

Amsterdam University Medical Centres

Vrije Universiteit Amsterdam

De Boelelaan 1117

1081 HV Amsterdam

The Netherlands

12 August 2024, Amsterdam

Response to reviewers

Manuscript bmjopen-2023-083225 - "Optimized treatment of patients with enlarged Lateral lymph Nodes in Rectal Cancer: protocol of an international, multicentre, prospective registration study after extensive multi-disciplinary training (LaNoReC)"

Dear Research Editor of BMJ open, dear Dr. Royle,

Thank you for your time and effort to critically review our manuscript "Optimized treatment of patients with enlarged Lateral lymph Nodes in Rectal Cancer: protocol of an international, multicentre, prospective registration study after extensive multi-disciplinary training (LaNoReC)" and for providing valuable feedback. We highly appreciate the opportunity to

revise our manuscript. The revisions are tracked using the track changes function or highlighted. Please see below for a point-by-point response to each of the reviewers' comments and suggestions.

Yours sincerely,

Dr. M. Kusters

On behalf of the co-authors; EGM van Geffen and the LaNoReC study group

Reviewer: 1

1. In describing the study outcome, both DFS and OS are suggested as outcome measures. Why did you not just go for OS? One could argue that OS alone would sufficient and a more robust outcome measure especially when introducing lateral lymph node dissection which might increase the number of surgical adverse events.

A: Thank you for reviewing this manuscript. We believe that the dual assessment using both DFS and OS provides a more comprehensive and balanced evaluation of the impact of LLND in our study population. While OS is indeed a more robust and definitive outcome measure, it may not fully reflect the nuances of disease progression, particularly in a population at older age with competing risks of death. Therefore, we have chosen to also report DFS to evaluate effectiveness of LLND in preventing disease recurrence, as combined measure of local- and distant recurrence and survival.

2. The protocol suggests re-staging MRI to be optional. Why did you not plan to make re-staging MRI mandatory in the protocol? I understand that the surgical indication will not be affected, however, systematic re-staging MRI could possibly provide valuable additional information to this otherwise ambitious project.

A: Thank you very much for your suggestion regarding making re-staging MRI mandatory in our protocol. We agree that systematic re-staging MRI could provide valuable additional information to the study. However, due to the nature of this being a registry study, it is not feasible to require a mandatory re-staging MRI.

That said, it is important to note that re-staging MRI after neoadjuvant (chemo)radiation is already a standard practice in all currently participating LaNoReC centers. We expect that nearly all patients will undergo re-staging MRI before surgery, allowing us to gather the valuable data you mentioned without formally mandating it in the study protocol. This has been clarified in the method section on page 10, lines 14-17 (see track changes).

3. Several neoadjuvant regimes will be used. Do you expect a difference in oncological effect on lateral nodes depending on given CRT or RAPIDO-style therapy? Would it be interesting to perform a pre-defined subgroup analysis of this? Would such a analysis be adequately "powered" in your opinion based on the expected distribution between CRT/RAPIDO?

A: Thank you for your insightful comment. We anticipate that roughly half of the patients will receive standard CRT, while the other half will undergo alternative neoadjuvant therapies, such as RAPIDO or more extensive regimens involving additional systemic therapy. After three years of follow-up, we expect minimal differences in the oncological effects on LLR rates between these treatment approaches. While a subgroup analysis of the various neoadjuvant therapies would indeed be valuable, the expected small differences between these treatments may not provide sufficient statistical power for meaningful sub-analysis. Consequently, we have decided not to include these subgroup analyses in the protocol.

4. Please define LLND more clearly in the protocol. I think you are going to use en-bloc compartment dissection instead of node-picking? If so please describe.

A: Thank you very much for this comment. Indeed, en-bloc compartment dissection will be used instead of node-picking procedures. A more detailed description of the procedure has been added to page 12, lines 11-18 (see track changes).

5. How will patients in which complete lymph node dissection is not done/possible be managed during follow-up and described? Exclusion from study?
A: Inclusion in the study is determined by the primary MRI according to the inclusion and exclusion criteria outlined in the manuscript (page 9, lines 14-20). If an included patient does not undergo nerve-sparing, minimally invasive LLND due to more extensive disease, complete clinical response, or the decision from a clinician or patient to refrain from LLND, the patient will still remain in the study for the purpose of collecting oncological outcomes. However, these patients will not be included in the main analysis. This process is described in detail on page 12, lines 1-9 (see highlighted section).

6. Which type of surgical resections will be included and in which expected numbers? Lateral lymph nodes are more common in medium/low rectal cancer. It might be expected that a sizeable proportion will undergo an APR with permanent colonostomy. Could you provide expected numbers and discuss this in relation to your planned follow-up with LARS-questionnaires?

A: Thank you for this comment. Indeed, we expect that a significant proportion of patients (between 50-70%) will require an APR due to distal location or sphincter involvement of the primary tumor. For those undergoing an APR or TME with temporary or permanent stoma, we will focus on collecting quality of life questionnaires, and not distribute LARS-

questionnaires. This has been added to page 13, lines 7-8 (see track changes).

7. You expect a drop-off of 5% and have sized the study cohort thereafter. What do you base 5% on. Would i be reasonable to increase the cohort expectation a 10% drop-off?

A: Thank you for the suggestion. We have implemented several strategies to minimize drop-off, including comprehensive follow-up procedures. Patients enrolled in specialized studies with multidisciplinary care tend to adhere more strictly to follow-up schedules, contributing to a lower drop-off rate. Additionally, centers conducting the follow-up are typically open for data collection. If a center is not open, the informed consent form allows us to request follow-up data from those centers. Moreover, patients who do not undergo neve-sparing minimally invasive LLND, will still be included in the study, but in the registration arm rather than the primary arm. These measures are designed to enhance patient retention and adherence, thereby supporting our assumption of a 5% drop-off.

8. You are planning an interim-analysis. Please clearly define how this will be performed and which parameters this will include. Please also define under which circumstances the interim analysis could prompt the study to be closed ahead of time.

A: Thank you for the comment. The interim analysis will focus on LLR-rate and short term complications and functional outcomes. This, together with the consideration for early closure of the study has been added to page 15, lines 12-19 (see track changes).

Reviewer: 2

1. Evaluation of surgical procedure and standardization is key to success in the trials for surgical procedures. In your study, you have planned that LLNDs will be performed by a select team of surgeons after completing adequate human cadaver training, intensive proctoring and assessment of procedural videos. Are there any evaluating tools or organized check-list in your protocol to guarantee the performance of well-trained surgeon or standardization of the procedures?

A: Thank you for your time for reviewing this manuscript. We fully agree on the importance of standardization. To ensure high-quality performance, our protocol includes comprehensive cadaver training, intensive proctoring, and detailed assessment of procedural videos. We have now provided a more detail of the LLND protocol on page 12, lines 11-18 (see track changes). During the study, LLND surgeons will continue to be proctored for as long as deemed necessary by both the proctor and the surgeon. Additionally, to uphold a high standard of care, monthly video assessment feedback sessions are conducted, where all relevant patient videos are reviewed and discussed. While some variation in protocol may occur, it also reflects the inherent variability in surgical practice.

2. LLNDs can improve the oncologic outcomes, but might be harmful to functional outcomes, related to sexual or urinary functions. For secondary outcomes, do you have plan to evaluate specific functional outcomes about those?

A: We understand the concerns regarding functional controls. Therefore, we analyze functional outcomes including sexual and urinary dysfunction, as well as quality of life during follow-up. An statistical plan to analyze these specific secondary outcomes has been added to page 13, lines 21-23 (see track changes).

VERSION 2 - REVIEW

**Reviewer**	**2**
**Name**	**Ryoo, Seung-Bum**
**Affiliation**	**Seoul National University College of Medicine, Surgery**
**Date**	**29-Aug-2024**
**COI**	**no**

I have reviewed the manuscript of 'Optimized treatment of patients with enlarged lateral lymph nodes in rectal cancer: protocol of an international, multicenter, prospective registration study after extensive multi-disciplinary training (LanoRec)'. This manuscript is about a study protocol for prospective registration study. This is a very important study and expected to get the significant results, but there are some points to be considered.

1. Evaluation of surgical procedure and standardization is key to success in the trials for surgical procedures. In your study, you have planned that LLNDs will be performed by a select team of surgeons after completing adequate human cadaver training, intensive proctoring and assessment of procedural videos. Are there any evaluating tools or organized check-list in your protocol to guarantee the performance of well-trained surgeon or standardization of the procedures?
2. LLNDs can improve the oncologic outcomes, but might be harmful to functional outcomes, related to sexual or urinary functions. For secondary outcomes, do you have plan to evaluate specific functional outcomes about those?
